# A Fast Calibration and Compensation Method for Magnetometers in Strap-Down Spinning Projectiles

**DOI:** 10.3390/s18124157

**Published:** 2018-11-27

**Authors:** Dafeng Long, Xiaoming Zhang, Xiaohui Wei, Zhongliang Luo, Jianzhong Cao

**Affiliations:** 1 School of Electronic Information and Electrical Engineering, Huizhou University, Huizhou 516007, China; longdafeng@nuc.edu.cn (D.L.); weixh0509@hzu.edu.cn (X.W.); hk3333@163.com (Z.L.); 2 Science and Technology on Electronic Test and Measurement Laboratory, North University of China, Taiyuan 030051, China; axiauto_gps@163.com

**Keywords:** magnetometer, calibration, ellipsoid fitting, spinning projectile

## Abstract

Attitude measurement is an essential technology in projectile trajectory correction. Magnetometers have been used for projectile attitude measurement systems as they are small in size, lightweight, and low cost. However, magnetometers are seriously disturbed by the artillery magnetic field during launch. Moreover, the error parameters of the magnetometers, which are calibrated in advance, usually change after extended storage. The changed parameters have negative effects on attitude estimation of the projectile. To improve the accuracy of attitude estimation, the magnetometers should be calibrated again before launch or during flight. This paper presents a fast calibration method specific for a spinning projectile. At the launch site, the tri-axial magnetometer is calibrated, the parameters of magnetometer are quickly obtained by optimal ellipsoid fitting based on a least squares criterion. Then, the calibration parameters are used to compensate for magnetometer outputs during flight. The numerical simulation results show that the proposed calibration method can effectively determine zero bias, scale factors, and alignment angle errors. Finally, a semi-physical experimental system was designed to further verify the performance of the calibration method. The results show that pitch angle error reduces from 3.52° to 0.58° after calibration. The roll angle error is reduced from 2.59° to 0.65°. Simulations and experimental results indicate that the accuracy of magnetometer in strap-down spinning projectile has been greatly enhanced, and the attitude estimation errors are reduced after calibration.

## 1. Introduction

Attitude measurement is an essential technology in projectile trajectory correction. Only a few types of attitude sensors are suitable for spinning projectiles since the sensors must withstand extremely large loads at launch and rapid spins during flight [1,2]. Magnetometers have been used for projectile attitude measurement systems as they are small in size, lightweight, and low cost [3]. The magnetometers are normally aligned with the body axes of the spinning projectile, and the attitudes of projectiles are calculated by the measured magnetic field information [4,5]. However, magnetometers are easily disturbed by external magnetic fields, and the magnetometer measurement outputs are corrupted by different kinds of error sources, e.g., bias errors, scale factors and misalignment errors [6,7,8]. Therefore, calibration of strap-down magnetometers must be performed to ensure their measurement accuracy [9]. The conventional calibration methods of magnetometer, such as the swinging method and the multi-position method [10], require external references or auxiliary equipment. To reduce the cost of the calibration procedures, many previous studies on calibration have focused on methods that require no external equipment [7,8,11,12], i.e., auto-calibration and the ellipsoid fitting method [12,13,14]. In addition, several researchers have proposed specific filtering methods for strap-down magnetometers [15], and an EKF filter is used to estimate the error parameters of magnetometers [16,17,18]. Since the filter models normally are nonlinear functions, nonlinear filter algorithms (EKF, UKF, and PF) are used to estimate the state of the system [19,20,21]. These calibration methods are effective for most applications, but they have several shortcomings such as the need for external equipment or auxiliary filter information is required.

Previous calibration studies have mainly focused on magnetic compasses, satellites, UAVs and various low-cost attitude measurement systems [17,22,23]. Note that these calibration methods are not suitable for projectile applications. First, the attitude measurement system is more seriously disturbed by the artillery magnetic field during launch [16]. In addition, the error parameters of the magnetometers, which are calibrated in advance, usually change after long duration storage, and the changed parameters will have negative effects on the attitude estimation of the projectiles. Therefore, to improve the accuracy of attitude estimation, the magnetometers must be calibrated again before launch or during flight. For weapon systems, a fast and simple calibration method is the most important. Thus, how to process a low cost calibration quickly and precisely remains a challenging problem for projectile attitude measurement systems. 

This paper aims to present a fast site calibration method specific for spinning projectiles. At the launch site, the tri-axial magnetometer is calibrated again, and the parameters of the tri-axial magnetometer including bias errors, scale factors and misalignment angles are obtained quickly by optimal ellipsoid fitting based on a least squares criterion. Then, these calibration parameters are used to compensate for magnetometer measured data during flight. The rest of this paper is organized as follows: Section 2 describes the magnetometer error model, and Section 3 discusses the calibration principle. Section 4 introduces numerical simulations, and Section 5 focuses on semi-physical experiments. The last section provides the conclusions.

## 2. Magnetometer Error Model

The measurement error of single-axis magnetometer mainly contains zero bias error and scale factor error, and the error model of single-axial magnetometer can be described as:

(1) $$H_{m}=(k_{n}+\delta k)H_{d}+H_{0}\approx k_{m}H_{d}+H_{0}$$

where, $k_{n}$ is ideal scale factor, δk is scale factor error; $k_{m}$ is actual scale factor, and $H_{0}$ is zero bias. $H_{m}$ is the magnetometer measured output. 

In general, a tri-axial magnetometer is used to measure the strength of Earth’s magnetic field. Therefore, the ideal error model of tri-axial magnetometer can be described as:

(2) $$\left\{ \begin{array}{l} H_{m1}=K_{1}H_{d}^{m}+H_{0}\\ K_{1}=k_{n}\left[\begin{array}{ccc} 1 & 0 & 0\\ 0 & 1 & 0\\ 0 & 0 & 1 \end{array}\right]+\left[\begin{array}{ccc} \delta k_{x} & 0 & 0\\ 0 & \delta k_{y} & 0\\ 0 & 0 & \delta k_{z} \end{array}\right]=\left[\begin{array}{ccc} k_{x} & 0 & 0\\ 0 & k_{y} & 0\\ 0 & 0 & k_{z} \end{array}\right] \end{array}\right.$$

where $K_{1}$ is a diagonal matrix, $k_{x}$, $k_{y}$ and $k_{z}$ indicate scale factors. $H_{d}^{m}={\left[H_{d,x}^{m},H_{d,y}^{m},H_{d,z}^{m}\right]}^{T}$ indicate ideal outputs of the tri-axial magnetometer.

**Figure 1:**
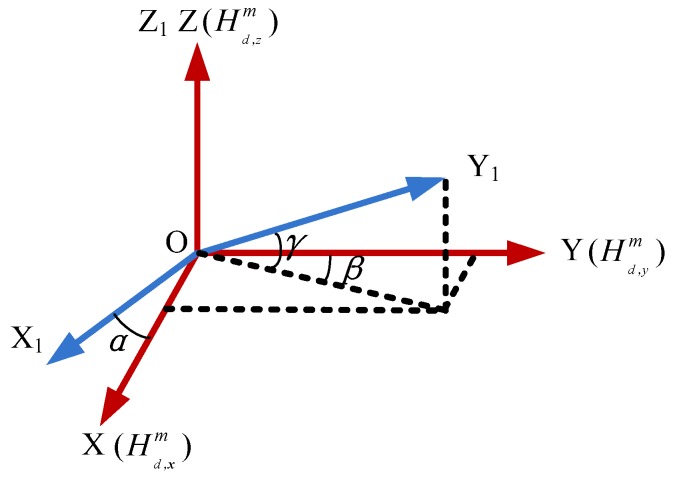
Non-orthogonal angles of magnetometers.

The magnetometers are normally aligned with the body axes of the projectile, but non-orthogonal angles are unavoidable. Figure 1 shows the non-orthogonal angles, where, O-XYZ defines the body coordinate frame, and O-X_1_Y_1_Z_1_ defines the sensor coordinate frame. Obviously, the magnetometers are not aligned with the body axes of the projectile. There are non-orthogonal angles (α,β,γ) between O-XYZ and O-X_1_Y_1_Z_1_. 

According to the vector relations shown in Figure 1, the non-orthogonal errors or misalignment errors which can be described as:

(3) $$\left\{ \begin{array}{l} H_{m2}=K_{2}H_{d}^{m}\\ K_{2}=\left[\begin{array}{ccc} \cos\alpha & 0 & \sin\alpha \\ \sin\beta \cos\gamma & \cos\beta \cos\gamma & \sin\gamma \\ 0 & 0 & 1 \end{array}\right] \end{array}\right.$$

If the non-orthogonal errors are taken into account, and the measurement error model of tri-axial magnetometer can be described as:

(4) $$\left\{ \begin{array}{l} H_{m}=K_{1}K_{2}H_{d}^{m}+H_{0}\\ \left[\begin{array}{c} H_{m,x}\\ H_{m,y}\\ H_{m,z} \end{array}\right]=\left[\begin{array}{ccc} k_{x} & 0 & 0\\ 0 & k_{y} & 0\\ 0 & 0 & k_{z} \end{array}\right]\left[\begin{array}{ccc} \cos\alpha & 0 & \sin\alpha \\ \sin\beta \cos\gamma & \cos\beta \cos\gamma & \sin\gamma \\ 0 & 0 & 1 \end{array}\right]\left[\begin{array}{c} H_{d,x}^{m}\\ H_{d,y}^{m}\\ H_{d,z}^{m} \end{array}\right]+\left[\begin{array}{c} H_{0,x}\\ H_{0,y}\\ H_{0,z} \end{array}\right] \end{array}\right.$$

According to magnetometer measurement error model (4), the compensation model of the tri-axial magnetometer can be described as:

(5) $$\left\{ \begin{array}{l} H_{d}^{m}=K_{2}^{-1}K_{1}^{-1}(H_{m}-H_{0})=K^{-1}(H_{m}-H_{0})\\ K^{-1}=\left[\begin{array}{ccc} \sec\alpha /k_{x} & 0 & -\tan\alpha /k_{z}\\ -\sec\alpha \tan\beta /k_{x} & \sec\beta \sec\gamma /k_{y} & \left(-\sec\beta \tan\gamma +\sec\alpha \tan\beta \right)/k_{z}\\ 0 & 0 & 1/k_{z} \end{array}\right] \end{array}\right.$$

To compensate the magnetometer measured outputs, the error parameters in $H_{0}$, matrix K, and inverse of matrix K ($K^{-1}$) must be determined. Since the non-orthogonal angles (α,β,γ) normally are small angles (cosi≈1,sinj≈j), and the matrix $K_{2}$ in Equation (3) can be simplified as follows:

(6) $$K_{2}\approx \left[\begin{array}{ccc} 1 & 0 & \alpha \\ \beta & 1 & \gamma \\ 0 & 0 & 1 \end{array}\right]$$

Substituting the approximate matrix of (6) into error model (4), the tri-axial magnetometer error model (4) can be simplified the follows:

(7) $$\left[\begin{array}{c} H_{m,x}\\ H_{m,y}\\ H_{m,z} \end{array}\right]=\left[\begin{array}{ccc} k_{x} & 0 & \alpha k_{x}\\ \beta k_{y} & k_{y} & \gamma k_{y}\\ 0 & 0 & k_{z} \end{array}\right]\left[\begin{array}{c} H_{d,x}^{m}\\ H_{d,y}^{m}\\ H_{d,z}^{m} \end{array}\right]+\left[\begin{array}{c} H_{0,x}\\ H_{0,y}\\ H_{0,z} \end{array}\right]$$

Similarly, by substituting inverse of matrix (6) into (5), the tri-axial magnetometer compensation model (5) also can be simplified as follows:

(8) $$\left[\begin{array}{c} {\hat{H}}_{d,x}^{m}\\ {\hat{H}}_{d,y}^{m}\\ {\hat{H}}_{d,z}^{m} \end{array}\right]=\left[\begin{array}{ccc} 1/k_{x} & 0 & -\alpha /k_{z}\\ -\beta /k_{x} & 1/k_{y} & -\gamma /k_{z}\\ 0 & 0 & 1/k_{z} \end{array}\right]\left(\left[\begin{array}{c} H_{m,x}\\ H_{m,y}\\ H_{m,z} \end{array}\right]-\left[\begin{array}{c} H_{0,x}\\ H_{0,y}\\ H_{0,z} \end{array}\right]\right)$$

## 3. The Least Squares Ellipsoid Fitting 

The total intensity of Earth’s magnetic field that is measured by an ideal tri-axial magnetometer should be a constant scalar [12,24]. $\left\|H_{e}\right\|=\sqrt{{\left(H_{e,x}\right)}^{2}+{\left(H_{e,y}\right)}^{2}+{\left(H_{e,z}\right)}^{2}}=constant$, the $\left\|H_{e}\right\|$ indicates the total intensity of Earth’s magnetic field. Thus, according to the tri-axial magnetometer measurement error model (5), the total intensity of Earth’s magnetic field can be calculated:

(9) $${\left\|H_{e}^{m}\right\|}^{2}={\left(H_{e}^{m}\right)}^{T}H_{e}^{m}={\left(H_{m}-H_{0}\right)}^{T}{\left(K^{-1}\right)}^{T}\left(K^{-1}\right)\left(H_{m}-H_{0}\right)$$

Equation (9) indicates that the magnetic measured outputs are constrained to an ellipsoid or sphere [12]. Equation (9) can be rewrote as follows:

(10) $${\left(H_{m}\right)}^{T}\frac{{\left(K^{-1}\right)}^{T}K^{-1}}{{\left\|H_{e}^{m}\right\|}^{2}}H_{m}-2\frac{{\left(H_{0}\right)}^{T}{\left(K^{-1}\right)}^{T}\left(K^{-1}\right)}{{\left\|H_{e}^{m}\right\|}^{2}}H_{m}+\frac{{\left(H_{0}\right)}^{T}{\left(K^{-1}\right)}^{T}\left(K^{-1}\right)H_{0}}{{\left\|H_{e}^{m}\right\|}^{2}}=1$$

Equation (10) is an ellipsoid surface equation, but an actual tri-axial magnetometer normally has measurement errors, and magnetometer measured data can’t fit on a standard sphere. In general, the shape and origin of the sphere maybe changed. Here, the shifted origin indicates the existence of zero bias errors, and the changed shape indicates the existence of scale factor errors or misalignment angles. The equation of an ellipsoid can be described as [25]:

(11) $$F(\xi ,z)=\xi^{T}z=ax^{2}+by^{2}+cz^{2}+2dxy+2exz+2fyz+\ldots +2px+2qy+2rz+g=0$$

where, vector ξ defines ${\left[a,b,c,d,e,f,p,q,r,g\right]}^{T}$, and $z={\left[x^{2},y^{2},z^{2},2xy,2xz,2yz,2x,2y,2z,1\right]}^{T}$.

To ensure the procedure is optimal fitting, a least-squares criteria is used to the ellipsoid fitting:

(12) $$\underset{\xi \in R^{6}}{\min}={\left\|F(\xi ,z)\right\|}^{2}=\underset{\xi \in R^{6}}{\min}\xi^{T}D^{T}D\xi$$

where, the matrix D is given by:

(13) $$D=[\begin{array}{ccccc} x_{1}^{2} & y_{1}^{2} & z_{1}^{2} & 2x_{1}y_{1} & 2x_{1}z_{1}\ldots \\ x_{2}^{2} & y_{2}^{2} & z_{2}^{2} & 2x_{2}y_{2} & 2x_{2}z_{2}\\ \vdots & \vdots & \vdots & \vdots & \vdots \\ x_{N}^{2} & y_{N}^{2} & z_{N}^{2} & 2x_{N}y_{N} & 2x_{N}z_{N} \end{array}\begin{array}{ccccc} 2y_{1}z_{1} & 2x_{1} & 2y_{1} & 2z_{1} & 1\\ 2y_{2}z_{2} & 2x_{2} & 2y_{2} & 2z_{2} & 1\\ \vdots & \vdots & \vdots & \vdots & \vdots \\ 2y_{N}z_{N} & 2x_{N} & 2y_{N} & 2z_{N} & 1 \end{array}]$$

In order to obtain the optimal ellipsoid parameters, the ellipsoid fitting Equation (11) can be rewritten as follows:

(14) $${\left(X-X_{0}\right)}^{T}A(X-X_{0})=X^{T}AX-2X_{0}^{T}AX+X_{0}^{T}X_{0}=1$$

where, $A=\left[\begin{array}{ccc} a & d & e\\ d & b & f\\ e & f & c \end{array}\right]$ is the shape parameter matrix (3 × 3), $X_{0}=-A^{-1}\left[\begin{array}{c} p\\ q\\ r \end{array}\right]$ is the origin of the optimal ellipsoid.

In contrast to Equations (14) and (10), the following formulas can be obtained:

(15) $$\left\{ \begin{array}{l} KK^{T}=\frac{1}{{\left\|H_{e}^{m}\right\|}^{2}}A^{-1}\\ H_{0}=X_{0} \end{array}\right.$$

Using the simplified magnetometer error model (4), and the matrix operation ($KK^{T}$) can be calculated by:

(16) $$KK^{T}=\left[\begin{array}{lll} \left(\alpha^{2}+1\right)k_{x}^{2} & \left(\beta +\alpha \gamma \right)k_{x}k_{y} & \alpha k_{x}k_{z}\\ \left(\beta +\alpha \gamma \right)k_{x}k_{y} & \left(\beta^{2}+\gamma^{2}+1\right)k_{y}^{2} & \gamma k_{y}k_{z}\\ \alpha k_{x}k_{z} & \gamma k_{y}k_{z} & k_{z}^{2} \end{array}\right]$$

Therefore, the optimal ellipsoid parameter matrix A and ellipsoid origin X_0_ can be obtained by ellipsoid fitting, and the error parameters of the tri-axial magnetometer can be estimated by (15). 

**Figure 2:**
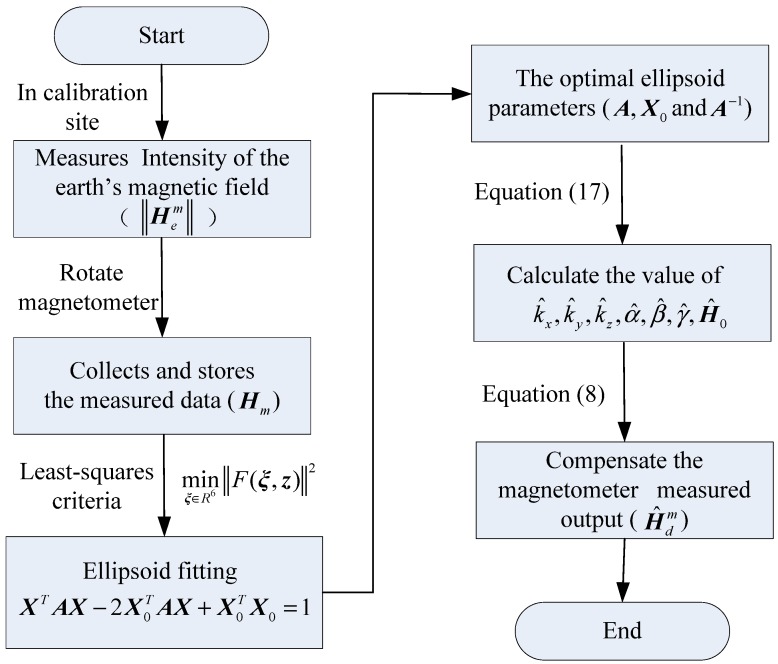
Algorithm flow chart for the calibration and compensation.

Assuming that the inverse matrix of A is $A^{-1}=\left[\begin{array}{lll} a^{\prime } & d^{\prime } & e^{\prime }\\ d^{\prime } & b^{\prime } & f^{\prime }\\ e^{\prime } & f^{\prime } & c^{\prime } \end{array}\right]$, and the error parameters of the tri-axial magnetometer (${\hat{k}}_{x},{\hat{k}}_{y},{\hat{k}}_{z},\hat{\alpha },\hat{\beta },\hat{\gamma },{\hat{H}}_{0}$) can be calculated by:

(17) $$[\begin{array}{l} {\hat{k}}_{x}=\frac{\sqrt{a^{\prime }c^{\prime }-{e^{\prime }}^{2}}}{\sqrt{c^{\prime }}\left\|H_{e}^{m}\right\|}\\ {\hat{k}}_{y}=\frac{\sqrt{\left(b^{\prime }-{f^{\prime }}^{2}\right)\left(a^{\prime }{c^{\prime }}^{2}-c^{\prime }{e^{\prime }}^{2}\right)-{\left(c^{\prime }d^{\prime }-e^{\prime }f^{\prime }\right)}^{2}}}{\left\|H_{e}^{m}\right\|\sqrt{a^{\prime }{c^{\prime }}^{2}-c^{\prime }{e^{\prime }}^{2}}}\\ {\hat{k}}_{z}=\sqrt{c^{\prime }}/\left\|H_{e}^{m}\right\|\\ \hat{\alpha }=e^{\prime }/\sqrt{a^{\prime }c^{\prime }-{e^{\prime }}^{2}}\\ \hat{\beta }=\frac{c^{\prime }d^{\prime }-e^{\prime }f^{\prime }}{{\hat{k}}_{y}\left\|H_{e}^{m}\right\|\sqrt{a^{\prime }{c^{\prime }}^{2}-c^{\prime }{e^{\prime }}^{2}}}\\ \hat{\gamma }=\frac{f^{\prime }}{{\hat{k}}_{y}\left\|H_{e}^{m}\right\|\sqrt{c^{\prime }}}\\ {\hat{H}}_{0}=X_{0} \end{array}$$

where, $\left\|H_{e}^{m}\right\|$ is the total intensity of Earth’s magnetic field in the calibration site. Therefore, the algorithm flow chart for the calibration and compensation is shown in Figure 2.

## 4. Algorithm Performance 

This section describes how numerical simulations assess the performance of the proposed magnetometer calibration method based on ellipsoid fitting. The section is shown by considering the calibration of magnetometers in two different error sources. Table 1 lists the magnetometer error parameters assumed in the simulation. As can be seen from Table 1, the difference between two magnetometers is that the magnetometer (case 2) is affected by strong magnetic interference, and has large error parameters. In the numerical simulation, firstly, the ideal outputs are generated by the ideal model of tri-axial magnetometer. Then, according to the error model of the magnetometer, the actual measurement outputs including bias errors, scale factor errors and alignment angle errors are generated. Here, the International Geomagnetic Reference Model generates the local magnetic field of the Earth in the simulation.

**Figure 3:**
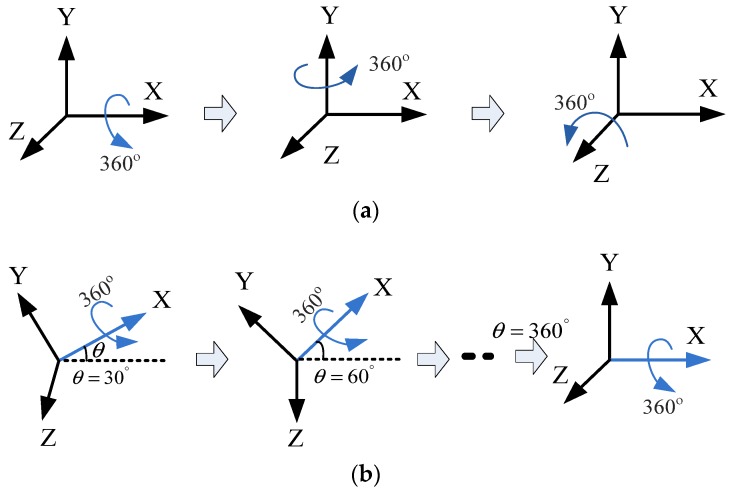
Calibration attitude changes. (**a**) Attitude change 1; (**b**) Attitude change 2.

Calibration of the tri-axial magnetometer is carried out according to the following four different attitude changes, as follows: (1) At horizontal state, as shown in Figure 3a, each axis of a tri-axial magnetometer successively spins 360°. (2) One axis of the tri-axial magnetometer successively spins 360° under different inclination angles, as shown in Figure 3b, the magnetometer successively spins 360° around X-axis when the inclination angles are 30°, 60°, and 90°, etc. (3) The magnetometer spins 360° at the same time. (4) The magnetometer randomly rotates in various directions.

**Figure 4:**
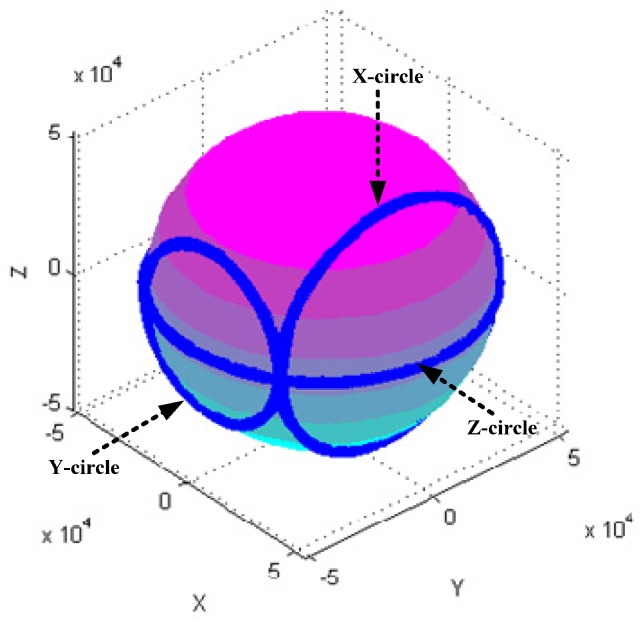
Optimal ellipsoid fitting (EF1).

**Figure 5:**
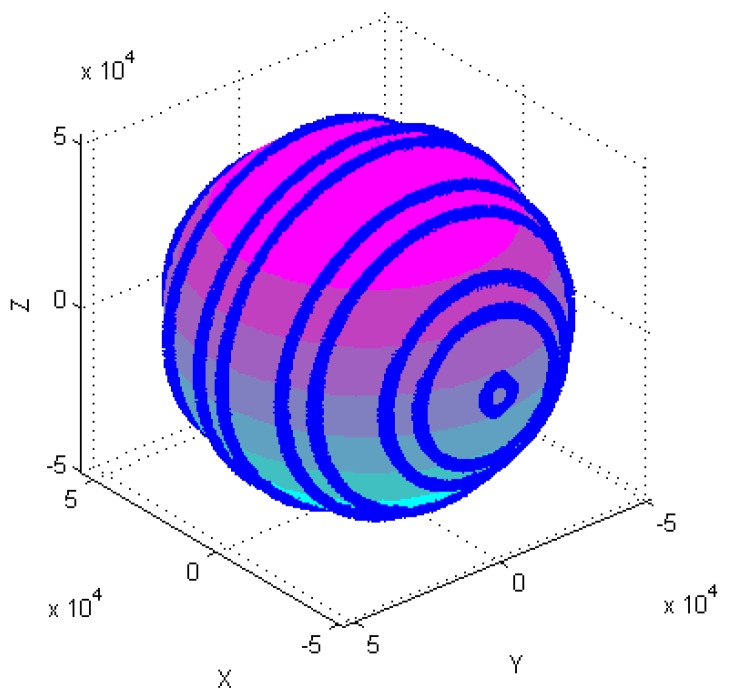
Optimal ellipsoid fitting (EF2).

**Figure 6:**
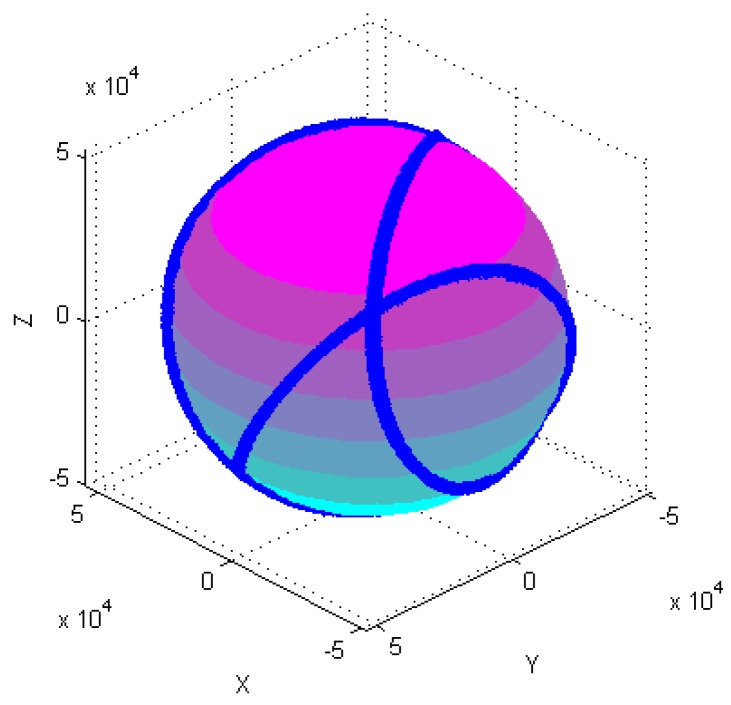
Optimal ellipsoid fitting (EF3).

**Figure 7:**
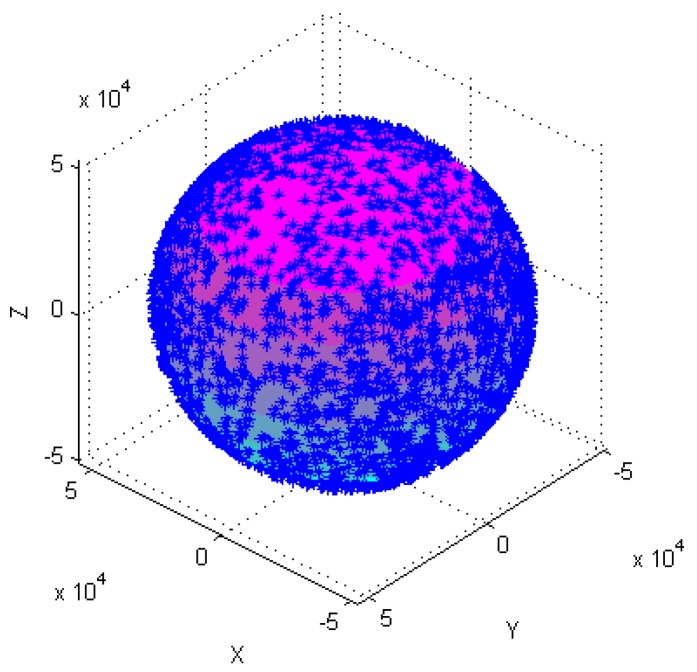
Optimal ellipsoid fitting (EF4).

**Figure 8:**
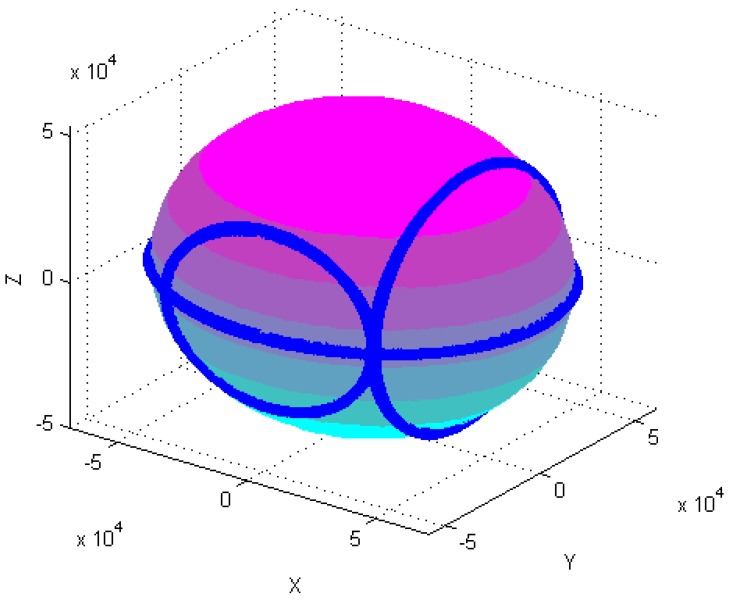
Optimal ellipsoid fitting (EF5).

**Figure 9:**
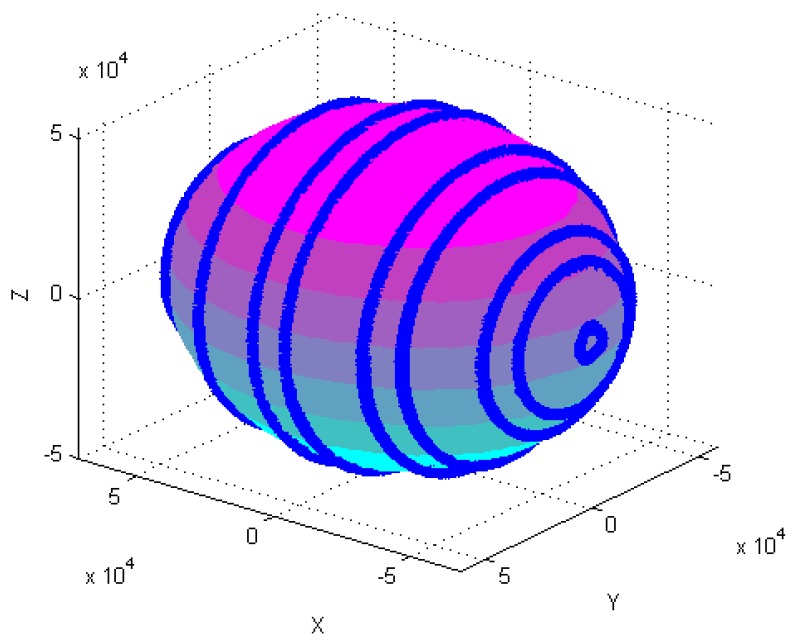
Optimal ellipsoid fitting (EF6).

**Figure 10:**
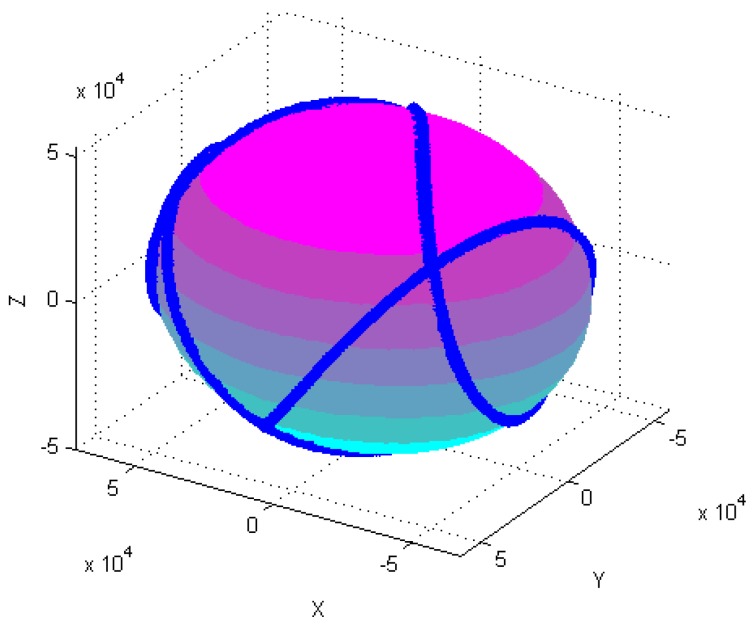
Optimal ellipsoid fitting (EF7).

**Figure 11:**
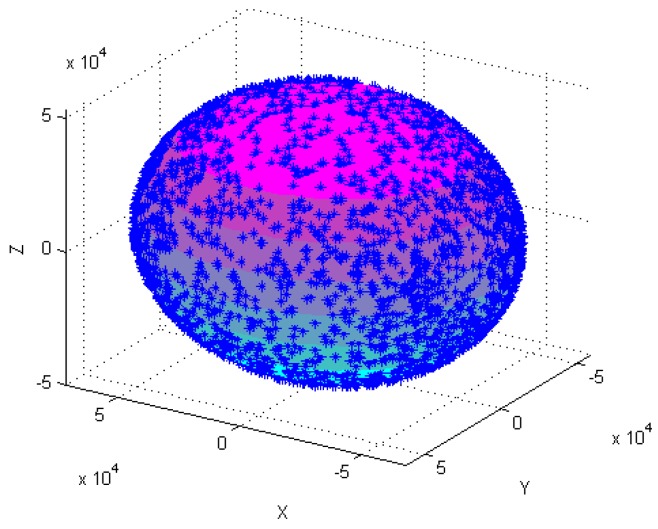
Optimal ellipsoid fitting (EF8).

Figure 4, Figure 5, Figure 6, Figure 7, Figure 8, Figure 9, Figure 10 and Figure 11 show optimal ellipsoid fitting results. The ellipsoid is obtained by the ellipsoid fitting method described in this paper. Here, the blue points of the sphere surface indicate measured magnetic data. For example, in Figure 4, the magnetometer calibration attitude change is the same as change (1), the x-circle, y-circle and z-circle indicate magnetometer measured data when each axis spins 360°, respectively. As shown in Figure 5, Figure 6 and Figure 7, these calibration attitudes correspond to the change (2)–(4), respectively. In Figure 7, it can be seen that the magnetometer measured data almost fits on an ellipsoid, which is consistent with the previous conclusion. 

Table 2 shows the calibration results. It can be seen that magnetometers can be calibrated by four different attitude changes. In case1, the ellipsoid fitting results correspond to the EF1–EF4. Where, there are minimal estimation errors in EF4. On the contrary, the EF1 has maximal estimation errors. In the case 2, the ellipsoidal fitting results show in Figure 8, Figure 9, Figure 10 and Figure 11. Because the magnetometer have large error parameters, the ellipsoid fitting results (EF5–EF8) are obviously different from case 1 (EF1–EF4). It can be seen that the EF8 calibration precision is the highest, and the EF5 has the maximal error. The numerical simulation results show that the proposed calibration method can effectively determine zero bias, scale factors, and alignment angle errors. Moreover, if the measured data can cover the whole ellipsoid surface such as EF4 and EF8, the calibration result is optimal.

**Figure 12:**
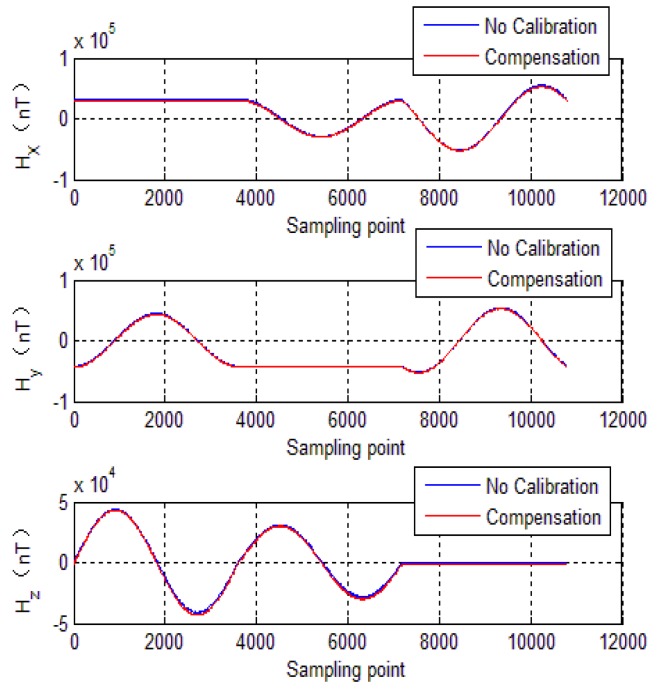
Magnetometer measured outputs.

**Figure 13:**
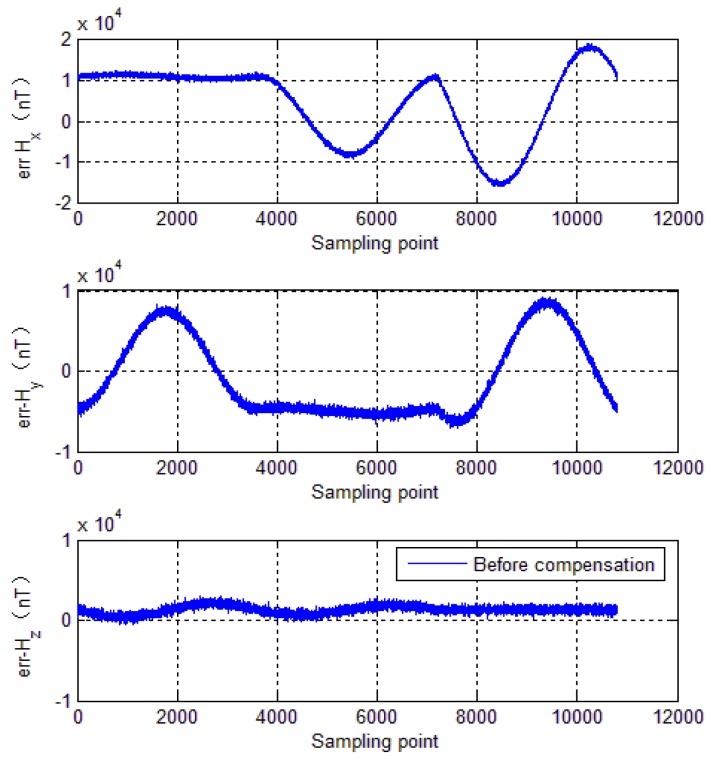
Measurement error before calibration.

**Figure 14:**
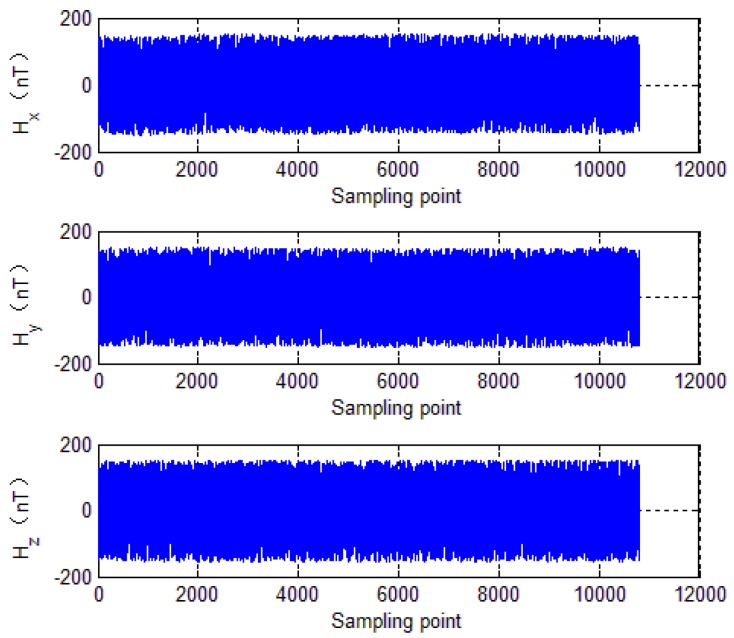
Measurement error after compensation.

**Figure 15:**
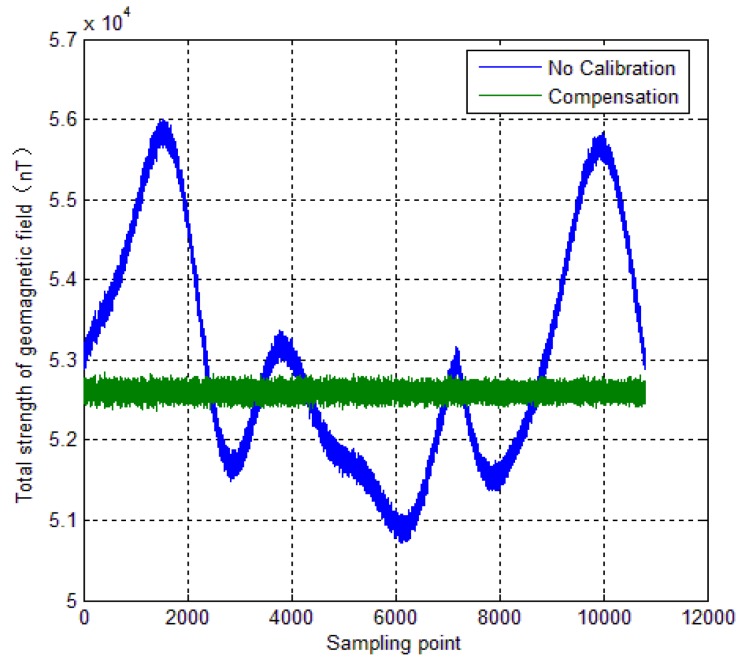
Total intensity of the magnetic field.

To further evaluate calibration algorithm performance, the calibration parameters of the EF4 are used to compensate the tri-axial magnetometer measured outputs. The compensation results are shown in Figure 12, Figure 13, Figure 14 and Figure 15, where, Figure 12 shows tri-axial magnetometer measured outputs before and after compensation. Figure 13 shows the magnetometer measurement error curve before calibration, while Figure 14 shows the magnetometer measurement error curve after compensation. 

It can be seen that without compensation, the each axis error standard deviation is 1440 nT, 890 nT and 360 nT, respectively, and the errors reduce to less than 200 nT after compensation. Figure 15 shows total intensity of Earth’s magnetic field before and after calibration. It can be seen that without calibration, the total intensity of Earth’s magnetic field varies from 50,822 nT to 56,000 nT. However, after calibration, the total intensity of Earth’s magnetic field is approximately constant (52,600 nT). It can be seen that the calibration method brings a significant enhancement of the magnetometer measurement accuracy. The numerical simulations verify effectiveness of the calibration method mentioned in this paper. 

## 5. An Application in Spinning Projectile

**Figure 16:**
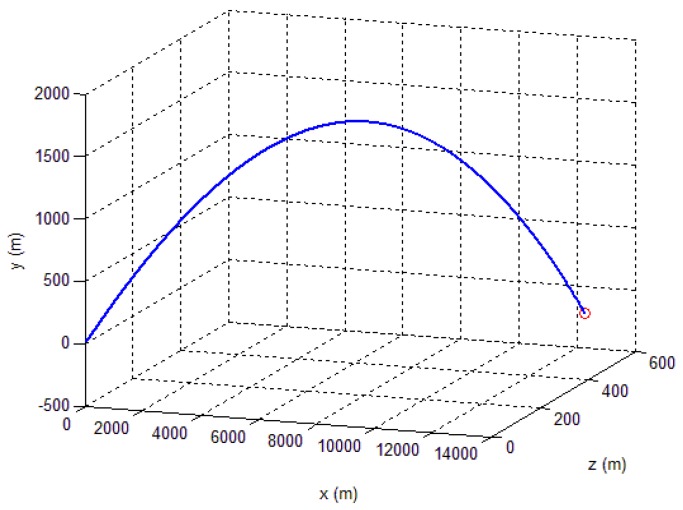
Trajectory of a spinning projectile.

**Figure 17:**
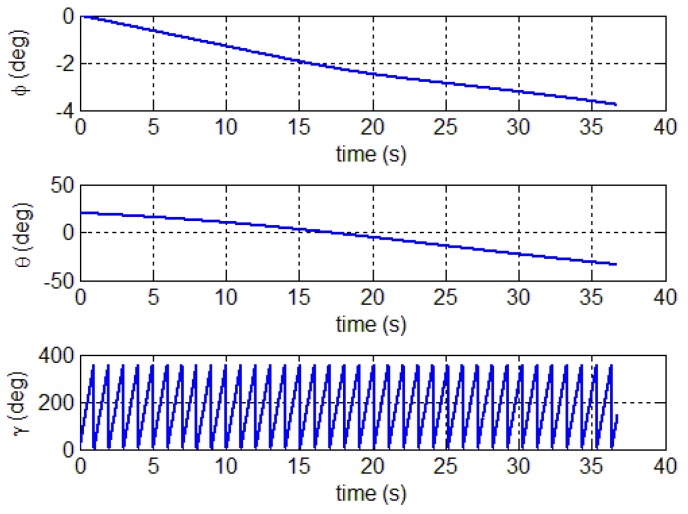
The full attitude of projectile.

In this section, the calibration method is used for a spinning projectile. The correction is experimentally assessed to evaluate the effectiveness of the calibration method in practice. Figure 16 shows the three-dimensional flight trajectory of the spinning projectile. The flight starting point is (0, 0, 0), and the end is marked by a red circle. Figure 17 shows the full attitude of the projectile. The simulated trajectory has 20° initial pitch angle and 0° initial yaw angle. The initial roll is random variables, and its initial roll rate is 720 deg s^−1^.

**Figure 18:**
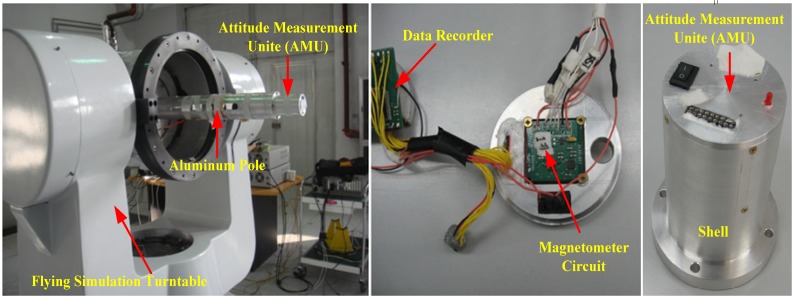
Semi-physical experimental system.

A semi-physical experimental system was designed for the simulation experiments. Figure 18 illustrates the homemade prototype. The experimental system is consist of two parts, one is attitude measurement unite (AMU) which contains a tri-axial magnetometer (HMC1043) circuit board, accelerometers and a data recorder, the other one is a flying simulation turntable. The flying simulation turntable can realize the simulation of any kind of projectile movement. To reduce the impact of turntable magnetic interference on the tri-axial magnetometer, the AMU is strap-down installed on a long aluminum pole (similar to a projectile), and the pole was fixed in the flying simulation turntable.

**Figure 19:**
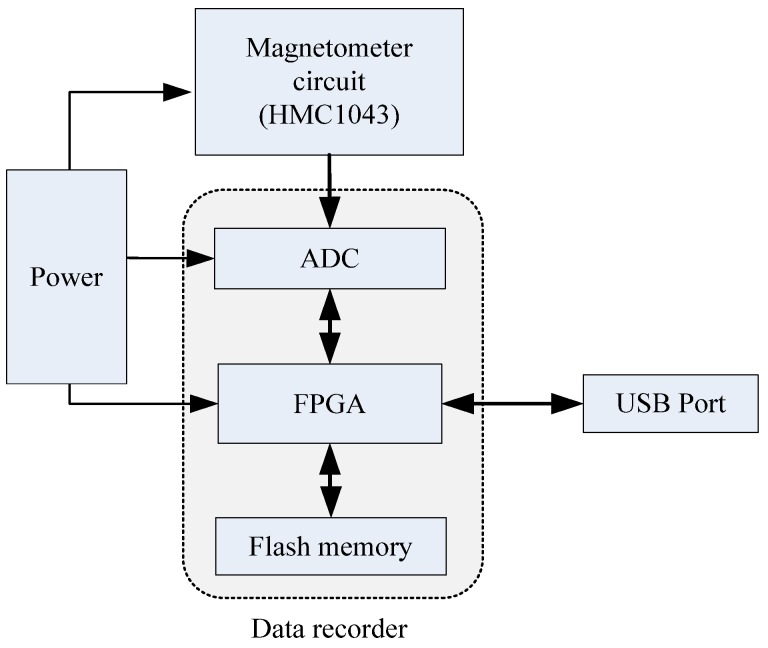
Hardware components diagram of the AMU.

The AMU is mainly comprised of tri-axial magnetometer circuit, data recorder, battery and protective shell. The hardware components diagram of the AMU is shown in Figure 19. The data recorder collects and stores the tri-axial magnetometer measurement output at a sampling frequency of 1000 Hz. Then, through the USB port, the magnetometer measured data was transferred to computer for attitude calculation.

**Figure 20:**
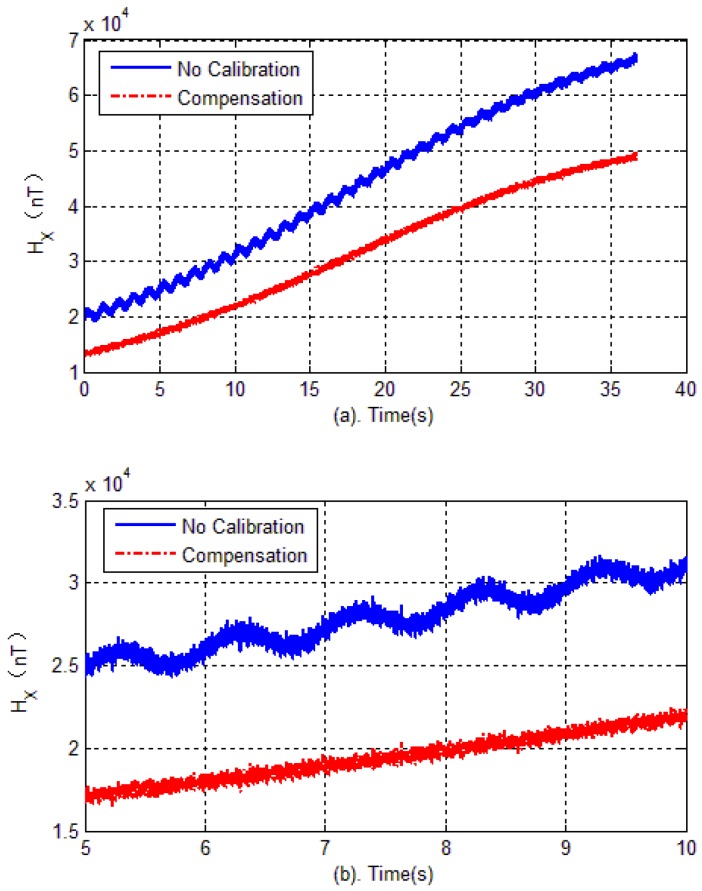
Magnetometer outputs of X-axis.

**Figure 21:**
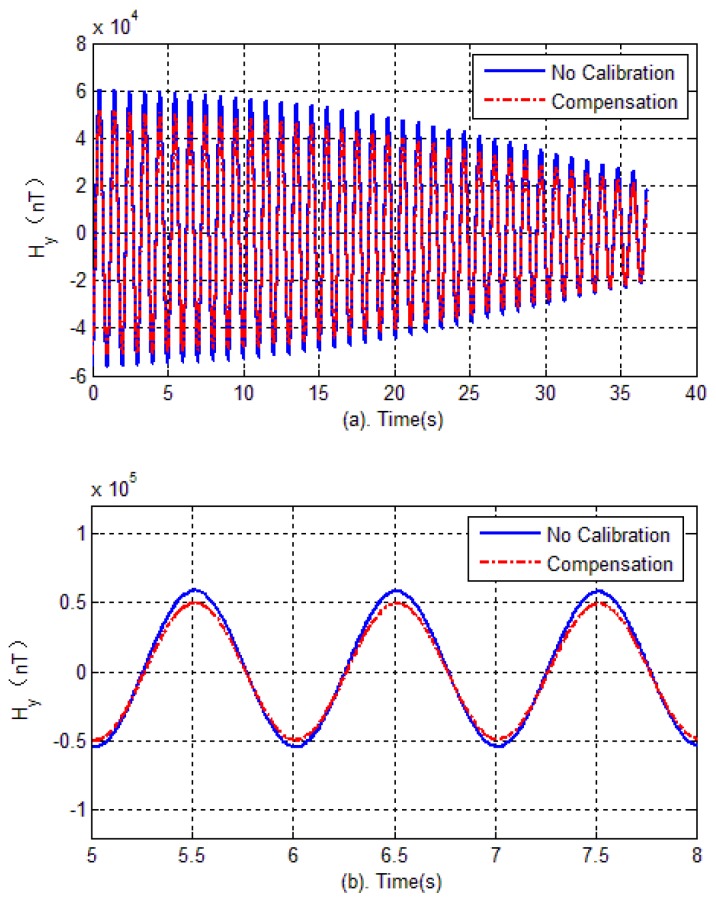
Magnetometer outputs of Y-axis.

**Figure 22:**
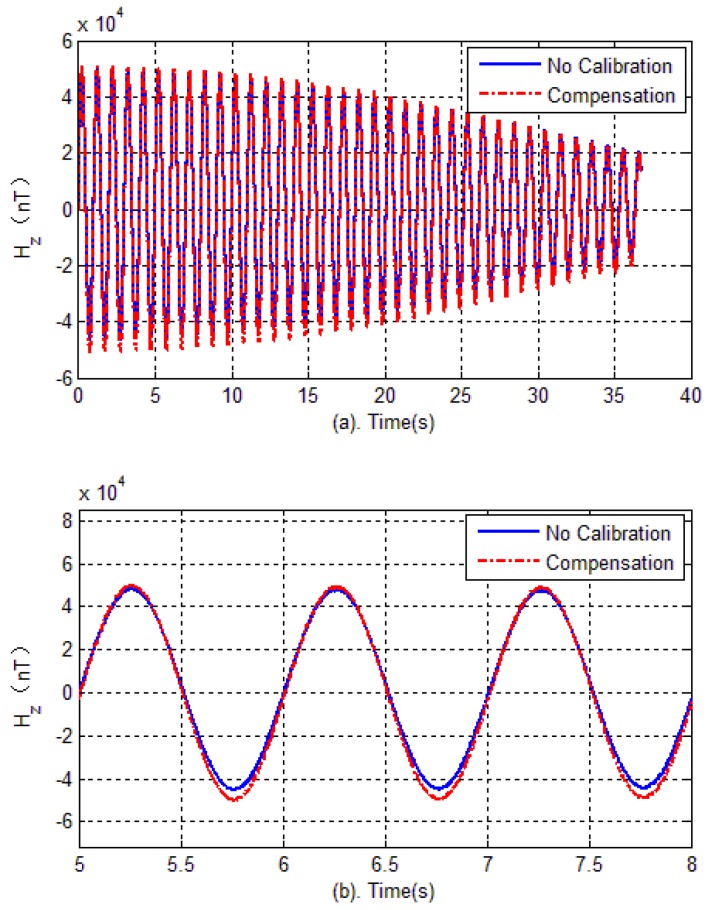
Magnetometer outputs of Z-axis.

The experimental verification is carried out in two steps. Firstly, in the test field (projectile launch site), the tri-axial magnetometer is calibrated again. The calibration of the magnetometer is carried out by rotating the AMU in various directions, and the parameters of magnetometer including bias errors, scale factors and misalignment angles are quickly obtained by optimal ellipsoid fitting. These calibration parameters are used to compensate for the measured data during flight. Then, semi-physical simulation is carried out to verify the effectiveness of calibration in the launch site. The semi-physical simulation system simulates the attitude movement of the spinning projectile, and the AMU measured data is used to calculate the attitude of the projectile. Calibration results are shown in Figure 20, Figure 21 and Figure 22. Here, the solid blue lines represent each axis magnetometer measured outputs before calibration, the dotted red lines represent the each axis measured outputs after calibration. In order to see the details, the lower charts of each figures describe the local details in 5–8 seconds. 

**Figure 23:**
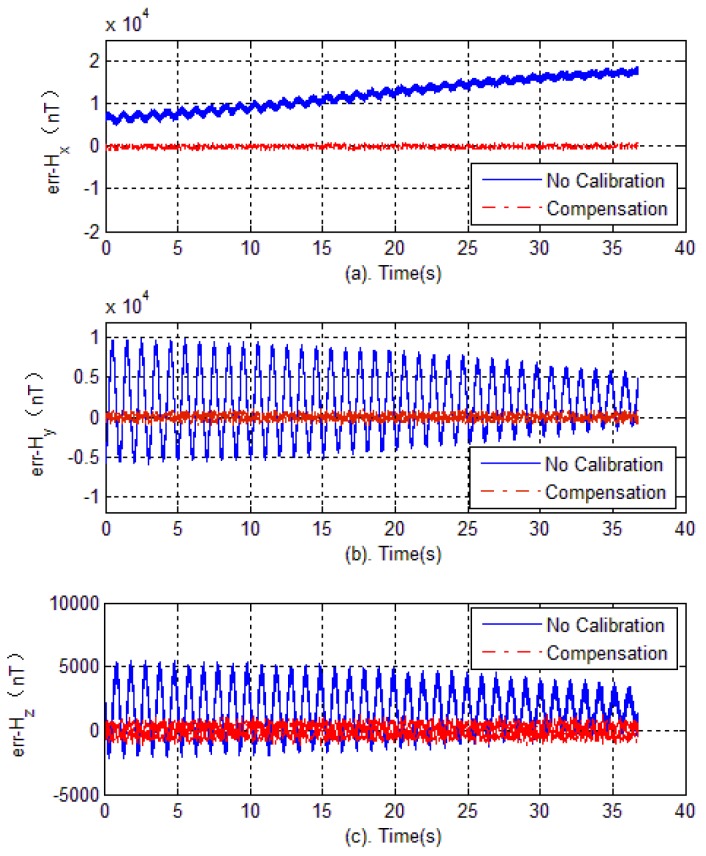
Outputs error versus time.

Figure 23 shows each axis measurement error of the tri-axial magnetometer. It can be seen that without calibration, the each axis measurement error (standard deviation) is 3513.4 nT, 4029.5 nT and 1743.5 nT, respectively. However, as expected, after performing the magnetometer calibration, the errors are reduced to less than 350 nT. The reduction of magnetic measurement errors comes from the compensation of magnetometer outputs.

**Figure 24:**
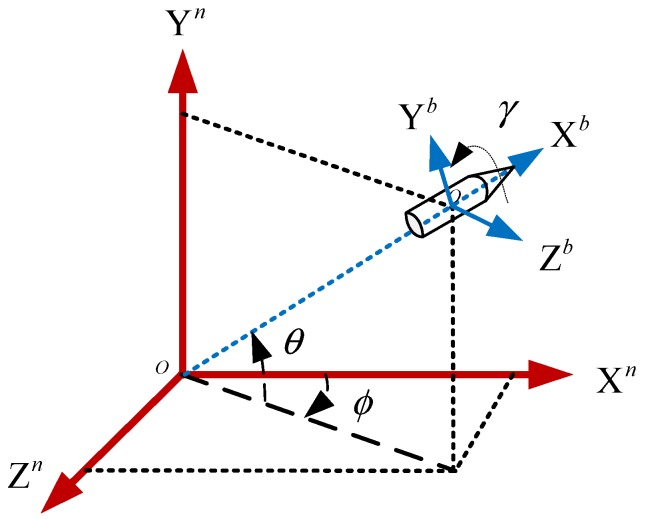
Definition of coordinate frame.

In order to further illustrate the calibration results, the attitude of spinning projectile is calculated using the magnetometer measured data before and after compensation. The definition of coordinate frame is shown in Figure 24. Where, the O-X^b^Y^b^Z^b^ defines body coordinate frame (b-frame), and O-X^n^Y^n^Z^n^ defines navigation coordinate frame (n-frame), the local level frame is selected as n-frame.

According to the definition of the Euler angle and rotation order, the directional cosine matrix is:

(18) $$C_{n}^{b}=\left[\begin{array}{ccc} \cos\phi \cos\theta & \sin\theta & -\cos\theta \sin\phi \\ \sin\gamma \sin\phi -\cos\gamma \cos\phi \sin\theta & \cos\gamma \cos\theta & \cos\phi \sin\gamma +\cos\gamma \sin\phi \sin\theta \\ \cos\gamma \sin\phi +\cos\phi \sin\gamma \sin\theta & -\cos\theta \sin\gamma & \cos\gamma \cos\phi -\sin\gamma \sin\phi \sin\theta \end{array}\right]$$

where ϕ, θ and γ represent yaw, pitch and roll angles, respectively. Therefore, the tri-axial magnetometer output is given by:

(19) $$H_{m}^{b}=C_{n}^{b}H_{e}^{n}$$

where $H_{m}^{b}={\left[H_{x}^{b},H_{y}^{b},H_{z}^{b}\right]}^{T}$ is the magnetic vector of the Earth in the b-frame, $H_{e}^{n}={\left[H_{x}^{n},H_{y}^{n},H_{z}^{n}\right]}^{T}$ is magnetic field vector of the Earth in the n-frame. 

Note that for projectile applications, if only the output information of the magnetometer is adopted for attitude estimation, the three attitude angles are not completely observable. Therefore, all the Euler angles cannot be estimated. To solve the problem, a common solution is the use of other auxiliary attitude sensor such as a gyroscope. Here, the yaw angle is assumed to be known (using the attitude feedback from the turntable), and the pitch and roll angles of the projectile can be calculated by:

(20) $$\left\{ \begin{array}{l} \theta =\arcsin\frac{H_{x}^{b}}{\sqrt{{\left(H_{x}^{n}\right)}^{2}+{\left(H_{y}^{n}\right)}^{2}}}-\arctan\frac{H_{x}^{n}\cos\phi -H_{z}^{n}\sin\phi }{H_{y}^{n}}\\ \gamma =\arcsin\frac{H_{z}^{n}}{\sqrt{{\left(H_{y}^{b}\right)}^{2}+{\left(H_{z}^{b}\right)}^{2}}}-\arctan\frac{H_{z}^{b}}{H_{y}^{b}} \end{array}\right.$$

**Figure 25:**
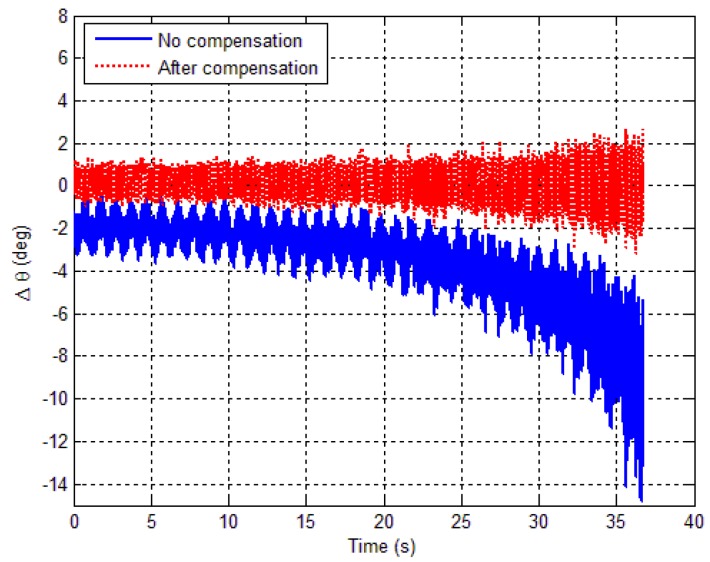
Pitch angle error versus time.

Therefore, substituting the magnetometer output into Equation (20), the pitch and roll angles are calculated by using the measured data. The pitch angle error is shown in Figure 25, where, the solid blue line represents the pitch angle error before correction, and the dotted red line represents the pitch angle error after correction. It can be seen that the attitude error increases with time.

The increase of the errors is due to the magnetic interference of the turntable. When the pitch angle is 20 °, the turntable magnetic interference is small. On the contrary, it is the largest at a −45° pitch angle. It can be seen that without compensation, the pitch angle error is 3.52° (1σ), and the maximum pitch error reaches 14.79°. However, after calibration, the pitch angle error reduces to 0.58° (1σ), and maximum pitch error reduces to more than 3.20°. 

**Figure 26:**
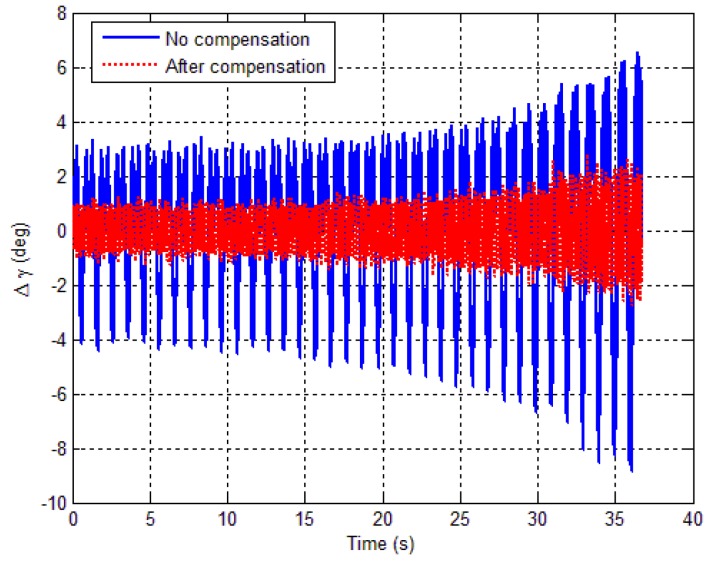
Roll angle error versus time.

Figure 26 shows the roll angle error before and after calibration. It can be seen that the roll angle error is 2.59° (1σ) before calibration, while the roll angle error reduces to 0.65° (1σ) after calibration. The maximum roll angle error reduces from 8.85° to less than 2.81°. Obviously, the attitude error reduction would have imposed significant positive effects on control performance of spinning projectiles. Simulation and experiment results show that the accuracy of the tri-axial magnetometer has been greatly enhanced, and the attitude estimation errors have been reduced after calibration. 

## 6. Conclusions

This paper presents a fast calibration method specific for a spinning projectile. At the launch site, the tri-axial magnetometer is calibrated again, and the error parameters of the magnetometer including bias errors, scale factors and misalignment angles are quickly obtained by optimal ellipsoid fitting based on a least squares criterion. Then, the calibration parameters are used to compensate the magnetometer outputs during flight. This method has the following advantages: First, using a least-squares fitting criteria to ensure the procedure is an optimal ellipsoid fitting. Second, without need for a precision turntable or other equipment, the calibration process is simple and easy to implement. Moreover, the method is also suitable for tri-axial accelerometers. Finally, a physical simulation system was designed to further verify the performance of the calibration method. The results show that pitch angle error is reduced from 3.52° to 0.58° (1σ) after calibration. Meanwhile, the roll angle error is reduced from 2.59° to 0.65° (1σ). Simulations and experimental results indicate that the accuracy of magnetometer in a strap-down spinning projectile has been greatly enhanced, and the attitude estimation errors of projectile have been reduced after calibration.

## Figures and Tables

**Table 1:** Error parameters of magnetometer assumed in the simulation.

Description	Parameters	X-axis	Y-axis	Z-axis
**Case 1** small error parameters	Scale factor	1.02	1.04	0.98
	Bias (nT)	505	430	580
	Alignment angle (″)	α	β	γ
		50	40	50
	Noise std (nT)	300	300	300
**Case 2** large error parameters	Scale factor	1.31	1.15	0.94
	Bias (nT)	2320	1830	1680
	Alignment angle (″)	α	β	γ
		60	40	60
	Noise std (nT)	810	660	920

**Table 2:** Calibration results.

Case	Ellipsoid Fitting (EF)	Scale Factor [Kx,Ky,Kz]	Bias (nT) [H_0x_,H_0y_,H_0z_]	Alignment Angle (″) [α,β,γ]
**Case1**	Real	1.0200,1.0400,0.9800	505.00,430.00,580.00	50.00,40.00,50.00
	EF1	1.0198,1.0400,0.9800	509.31,425.48,559.32	55.81,41.75,48.50
	EF2	1.0200,1.0400,0.9798	507.31,431.51,580.51	50.75,39.45,51.42
	EF3	1.0204,1.0397,0.9804	499.93,430.67,574.81	48.91,40.73,51.16
	EF4	1.0199,1.0399,0.9800	503.70,431.08,576.79	49.17,40.19,50.59
**Case2**	Real	1.3100,1.1400,0.9400	2320.00,1830.00,1680.00	60.00,40.00,60.00
	EF5	1.3098,1.1402,0.9397	2329.69,1832.03,1665.44	60.90,41.81,56.64
	EF6	1.3100,1.1389,0.9399	2322.27,1830.34,1681.36	60.85,38.94,61.33
	EF7	1.3100,1.1399,0.9400	2323.42,1829.94,1685.47	59.02,39.20,58.03
	EF8	1.3101,1.1400,0.9400	2321.39,1830.34,1679.08	59.67,40.75,60.92
